# Long‐term survival outcomes of patients with primary ocular adnexal MALT lymphoma: A large single‐center cohort study

**DOI:** 10.1002/cam4.5092

**Published:** 2022-07-30

**Authors:** Yuanzheng Liang, Rui‐ying Fu, Xue‐lin Liu, Xin‐di Liu, Ying‐shi Piao, Jian‐min Ma, Liang Wang

**Affiliations:** ^1^ Department of Hematology, Beijing Tongren Hospital Capital Medical University Beijing China; ^2^ Department of Pathology, Beijing Tongren Hospital Capital Medical University Beijing China; ^3^ Beijing Key Laboratory of Head and Neck Molecular Diagnostic Pathology Beijing China; ^4^ Beijing Tongren Eye Center, Beijing Ophthalmology and Visual Sciences Key Laboratory Beijing Tongren Hospital, Capital Medical University Beijing China

**Keywords:** immunochemotherapy, ocular adnexal MALT lymphoma, prognosis, radiation, surgery

## Abstract

**Background:**

Primary ocular adnexal extranodal marginal zone mucosa‐associated lymphoid tissue lymphoma (OAML) is a rare subtype of non‐Hodgkin's lymphoma, and no consensus has been defined concerning the optimal treatment strategies. This study aims to investigate the associations of disease characteristics and different treatments with long‐term outcomes of patients with localized OAML.

**Methods:**

A large retrospective cohort study was conducted in a single‐center of China, and 166 patients with newly diagnosed primary localized OAML were enrolled. Detailed data of disease characteristics at diagnosis and treatments were collected for all patients. We compared treatment response and progression‐free survival (PFS) among patients with different characteristics and treatments.

**Results:**

Of the 166 patients, 52 received complete resection of neoplasm, whereas 114 had residual lesion after surgery. Among the 114 patients, 61 underwent watchful waiting and 53 received further treatment including localized radiotherapy, chemotherapy, or combined radiotherapy and chemotherapy. Median follow‐up was 49 months. A total of 31 patients had disease progression or relapse, including four patients with such event more than five years after initial treatment. The 5‐year PFS was 73.9%, 70.6%, and 85.9%, whereas the 10‐year PFS was 69.3%, 59.2%, and 79.3%, among patients with complete resection of neoplasm, patients in the watchful waiting group and patients with further treatment, respectively. Patients with further treatment had longer PFS, compared with patients in the watchful waiting group (*p* = 0.011). Bilateral involvement at diagnosis was associated with significantly inferior PFS (*p* = 0.029), whereas age, IPI score, or TNM staging were not associated with PFS. No serious adverse reaction was reported among patients with further treatment.

**Conclusions:**

Bilateral involvement was associated with poor prognosis. Among patients with residual lesions after surgery, further treatment was associated with improved survival. Patients with OAML might experience disease progression or relapse more than five years after initial treatment.

## Graphical abstract

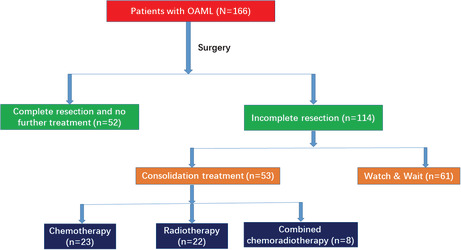

## 1 INTRODUCTION

Extranodal marginal zone lymphoma of mucosa‐associated lymphoid tissue (MALT lymphoma) is one type of low‐grade B cell non‐Hodgkin's lymphoma (NHL), and the most common pathological subtype of ocular adnexal lymphoma.^1^, ^2^ Ocular adnexal MALT lymphoma (OAML) refers to MALT lymphoma that primarily starts in ocular adnexal with lesions confined to the ocular adnexal, including conjunctiva, eyelid, orbit, and lacrimal gland, although a small number of patients also have extraocular involvement.^3^ So far, there is no consensus about optimal treatment strategies for OAML. Surgery is a common treatment modality for OAML, although relapse is a concern when no additional radiotherapy or chemotherapy is administered.^4^ Considering its indolent disease course, watchful waiting is also an acceptable option for both the patients and clinicians. Local radiotherapy is a preferred choice when treating OAML with localized lesions.^5^ However, ocular complications such as cataract and dry eyes are relatively common adverse events after radiotherapy, and the optimal dosage of radiotherapy is still under discussion.^6^ Patients with extraocular lesions are recommended to receive systemic treatment, including chemotherapy or immunochemotherapy.^7^ It remains however controversial whether systematic treatment should also be recommended to patients with localized lesions.

In this study, we compared long‐term outcomes of patients with OAML by different clinical characteristics and treatments, aiming to provide guidance for improved treatment of OAML, using data of 166 patients from a single institution in Beijing, China.

## 2 METHODS

### 2.1 Patient cohort

We enrolled a total of 166 patients with newly diagnosed OAML with lesions confined to the ocular adnexal, that is, without involvement of lymph nodes and other organs, from April 2008 to December 2021 in Beijing Tongren Hospital, China. Diagnosis of all patients was pathologically reviewed and confirmed through excised lesions or biopsied tissues, according to the 2016 revision of the WHO classification of tumors of hematopoietic and lymphoid tissues.^8^ Clinical characteristics were collected at the time of diagnosis for all patients, including results from physical examination, biochemical examination of blood, and imaging. We used the Ann Arbor Staging System and the American Joint Committee on Cancer (AJCC) TNM Staging System of OAML^9^ to determine the stage of all patients. Bilateral ocular adnexal involvement was defined as Ann Arbor stage I. We also studied the International Prognostic Index (IPI)^10^ as potential prognostic indicators for OAML. Patients with OAML who were not referred to the Department of Hematology and with unavailable detailed follow‐up information were excluded from our study.

### 2.2 Treatments

All patients first visited Beijing Tongren Eye Center and went through surgical treatment. The choice of surgical type, namely complete resection or partial resection (tumor biopsy) depends on the site of OAML and degree of infiltration, and the surgeon tried to remove the tumor without affecting the function and appearance of the eye. If the optic nerve was surrounded by tumor, only biopsy was done to confirm the diagnosis. Some patients had complete tumor resection, whereas other patients with postoperative residuals underwent watchful waiting or further treatment, including localized (i.e., radiotherapy alone) and systemic (i.e., chemotherapy alone, immunochemotherapy, chemotherapy plus radiotherapy, or immunochemotherapy plus radiotherapy) treatments, according to the involvement of lesions, severity of symptoms, and patient willingness.

For patients who received radiotherapy, three dimensional conformal radiotherapy (3DCRT) or intensity modulated radiotherapy (IMRT) were used at a median dose of 30 Gy (26–45 Gy). The dose of each fraction was 2 Gy, 5 times a week. The irradiation field includes the whole orbit of the affected eye. The cornea and lens were protected as much as possible during irradiation. Chemotherapy or immunochemotherapy including COP ± R (cyclophosphamide, vincristine and prednisolone±Rituximab), and CHOP ± R (cyclophosphamide, doxorubicine, vincristine, and prednisolone ± Rituximab), was administered every 21 days for four to six cycles. Two patients received FND ± R (fludarabine, mitoxantrone, and dexamethasone ± Rituximab) every 28 days for four cycles. Several patients received localized radiotherapy with a dose of 2600 ~ 3000 cGY in combination with chemotherapy or immunochemotherapy, including COP ± R or CHOP ± R, administered every 21 days for four cycles.

### 2.3 Treatment response and follow‐up

Interim treatment response was evaluated before the next course of treatment, and end of treatment response was assessed at 8 weeks after therapy. Follow‐up visits and evaluation of disease status were done every six months according to the revised response criteria for NHL,^11^ and classified as complete remission, partial remission, disease progression, and relapse through MRI or PET‐CT imaging. Each patient in our cohort had performed MRI assessments, and 89 patients had concurrent PET‐CT assessments. All patients were followed through telephone contact or inpatient or outpatient hospital visit until October 2021, with a median follow‐up of 49 (range: 2–156) months.

### 2.4 Statistical analyses

Progression‐free survival (PFS) was defined as the time from treatment initiation until disease progression, death from any cause, or last follow‐up, whichever came first. Overall survival (OS) was calculated for the entire patient group and defined as the time from treatment initiation until death from any cause, or last follow‐up, whichever came first. PFS and OS curves were constructed using the Kaplan–Meier method, and the statistical significance of the studied prognostic indicators was evaluated by log‐rank test. *p* < 0.05 was considered statistically significant. All analyses were performed using the SPSS 17.0.

### 2.5 Ethics statement

This study was approved by the Ethical Review Committee of Beijing Tongren Hospital, China. All procedures adhered to the tenets of the Declaration of Helsinki for research involving human subjects. The need for informed consent was waived because all patients had been de‐identified in our datasets.

## 3 RESULTS

### 3.1 Clinical characteristics

As is shown in Table 1, among the 166 patients, 97 were male, leading to a male‐to‐female ratio of 1.41. The median age at diagnosis was 57 years old (range: 19–89). Common symptoms of these patients included ocular foreign body sensation, eyelid edema, painless lump, exophthalmos, and impaired vision. One patient had B symptom (i.e., weight loss) and three patients had elevated serum level of lactate dehydrogenase (LDH). All patients were diagnosed as stage I OAML, with 36 patients having bilateral involvement. All but one patient was classified as low risk according to IPI. The most frequent involvement site was orbit (*N* = 47), followed by conjunctiva (*N* = 39), and lacrimal gland (*N* = 10). There were 12 patients with T4 involvement, including six patients with involvement of the local skin or subcutaneous tissue, five patients with involvement of optic nerve, and one patient with involvement of paranasal sinus.

**TABLE 1:** Clinical characteristics of the 166 patients with OAML

Clinical characteristics	No.	%
Sex		
Male	97	58.4
Female	69	41.6
Age, years		
≤60	90	54.2
>60	76	45.8
B symptom		
Yes	1	0.6
No	165	99.4
Lactate dehydrogenase		
Normal	163	98.2
Elevated	3	1.8
ECOG score		
0	130	78.3
1	36	21.7
Ann Arbor staging		
Stage I	166	100
IPI score		
0	88	53.0
1	77	46.4
≥2	1	0.6
Ocular involvement		
Unilateral	130	78.3
Bilateral	36	21.7
Anatomic site		
Conjunctiva	39	23.5
Orbit	47	28.3
Lacrimal gland	10	6.0
Eyelid	8	4.8
Complex	62	37.4
TNM stage		
(b) T1N0M0	39	23.5
(b) T2N0M0	69	41.6
(b) T3N0M0	46	27.7
(b) T4N0M0	12	7.2

### 3.2 Treatment, response, and follow‐up

**FIGURE 1:**
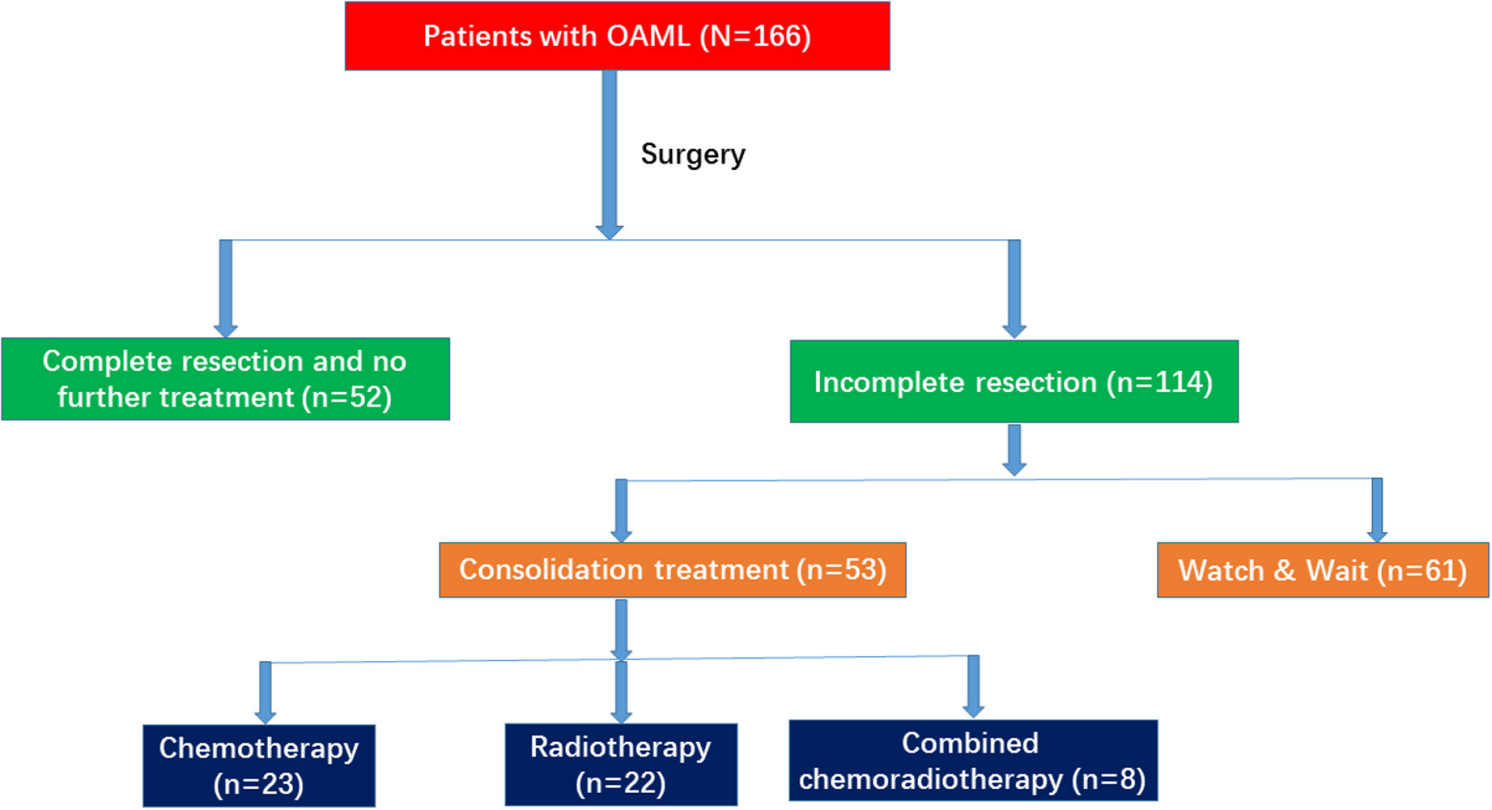
Treatment strategies of the 166 patients with primary OAML.

Fifty two patients had complete resection of neoplasm, whereas 114 had residual lesions after surgery (Figure 1). According to the involvement of lesions, severity of symptoms, and patient willingness, 61 patients underwent watchful waiting, whereas 53 received further treatment. Among 53 patients with further treatment, 22 received localized radiotherapy, and 23 received chemotherapy or immunochemotherapy, including COP ± R (*N* = 12), CHOP ± R (*N* = 9), and FND (*N* = 2). The other eight patients received localized radiotherapy in combination with chemotherapy or immunochemotherapy, including COP ± R (*N* = 4) and CHOP ± R (*N* = 4).

**FIGURE 2:**
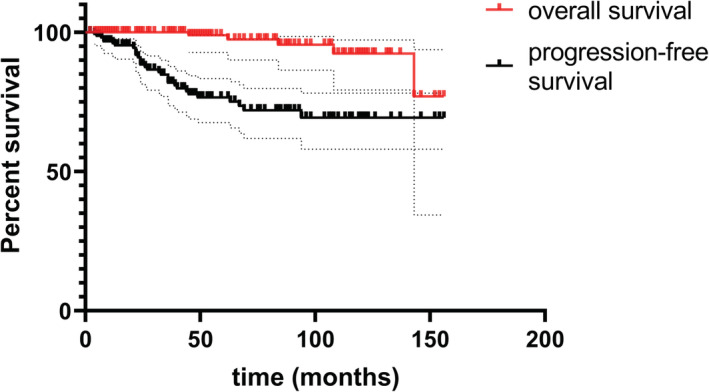
Survival outcomes of the whole cohort of patients with primary OAML. For the whole cohort, the 5 and 10‐year OS rates were 99% and 92%, respectively, whereas the 5 and 10‐year PFS rates were 76% and 69%, respectively.

Among the 166 patients, 86 had complete remission whereas 80 had partial remission (Table 2). A total of 31 patients had disease progression or relapse, including four patients with disease progression or relapse more than five years after initial treatment. The median remission time of all patients was 37 (range: 2–156) months. For the whole cohort, the 5 and 10‐year OS rates were 99% and 92%, respectively, whereas the 5 and 10‐year PFS rates were 76% and 69%, respectively (Figure 2).

**TABLE 2:** Treatment response of the 166 patients with OAML

Patient group	No.	Complete remission after treatment		Partial remission after treatment		Disease progression/relapse at last follow up	
		No.	%	No.	%	No.	%
Total	166	86	51.8	80	48.2	31	18.7
Complete resection of neoplasm	52	52	100.0	0	0.0	10	19.2
Watch & Wait	61	0	0.0	61	100.0	15	24.6
Consolidation treatment	53	34	64.2	19	35.8	6	11.3

All 52 patients with complete resection had complete remission (Table 2), with a median remission time of 40 (range: 2–154) months, although 10 of them had relapse, and the median time to progression was 24 (range: 4–67) months. The 5 and 10‐year PFS rates were 73.9% and 69.3% among patients with complete resection. All 61 patients with watchful waiting had partial remission, with a median remission time of 32 (range: 4–133) months, although 15 of them had disease progression, and the median time to progression was 24 (range: 5–69) months. The 5 and 10‐year PFS rates were 70.6% and 59.2% for patients with watchful waiting group. Among patients with complete resection or watchful waiting, the 25 patients that experienced disease progression or relapse were all local recurrence of ocular adnexa, whereas three of them had extraocular involvement including oral mucosa, elbow skin, and multiple deep lymph nodes.

Among the 53 patients that received further consolidation treatment, 34 had complete remission, whereas 19 had partial remission (Table 2). The median remission time was 49 (range: 4–156) months. Among these patients, two patients with complete remission experienced relapse and four patients with partial remission experienced disease progression during follow‐up, all in the primary site. The 5‐year and 10‐year PFS rates were 85.9% and 79.3% for patients in the further consolidation treatment group.

### 3.3 Survival, outcome, and prognostic factors

**FIGURE 3:**
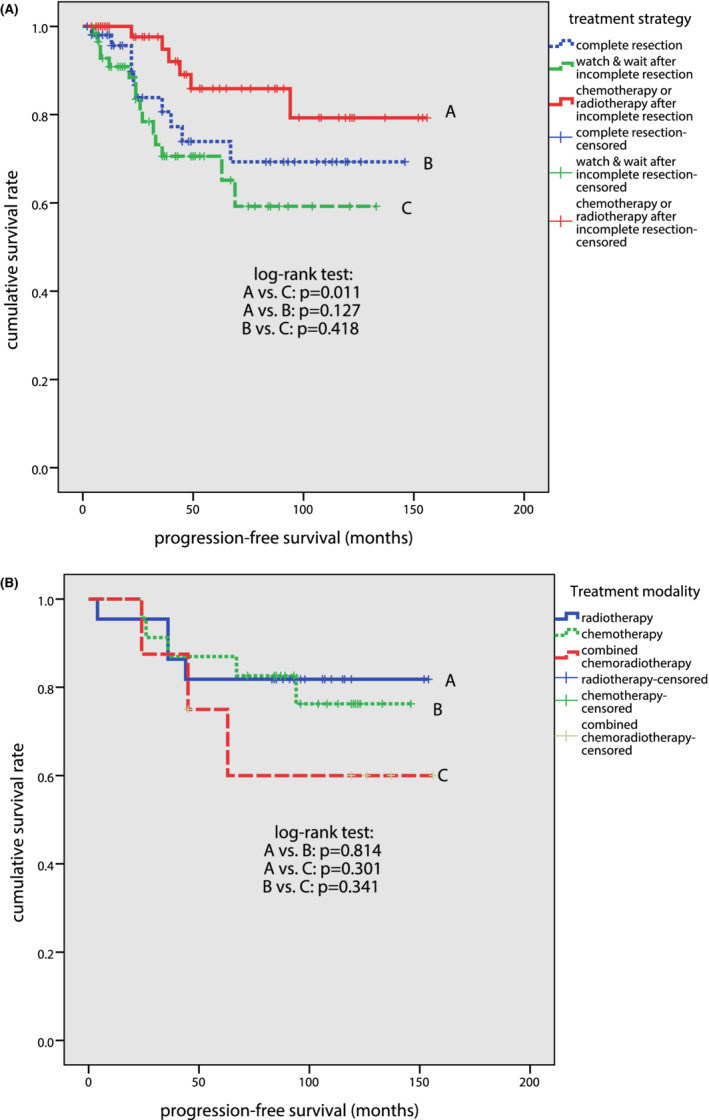
Survival curves for patients with primary OAML. (A) Progression‐free survival (PFS) curves among patients with complete resection, patients in the watchful waiting group, and patients in the further consolidation treatment group. (B) Progression‐free survival (PFS) curves among patients in the further consolidation treatment group: radiotherapy alone, chemotherapy alone, and combined with chemoradiotherapy.

Patients in the further consolidation treatment group had longer PFS, compared with patients in the watchful waiting group (*p* = 0.011) (Figure 3A). There was however no statistically significant difference between the PFS of patients with further treatment group and the PFS of patients with complete resection of neoplasm (*p* = 0.127). In the further consolidation treatment group, no statistically significant difference was noted in the PFS of patients with different treatment modalities (Figure 3B). No significant difference was found in the OS of patients with complete resection, watchful waiting, or further consolidation treatment (*p* = 0.414).

**FIGURE 4:**
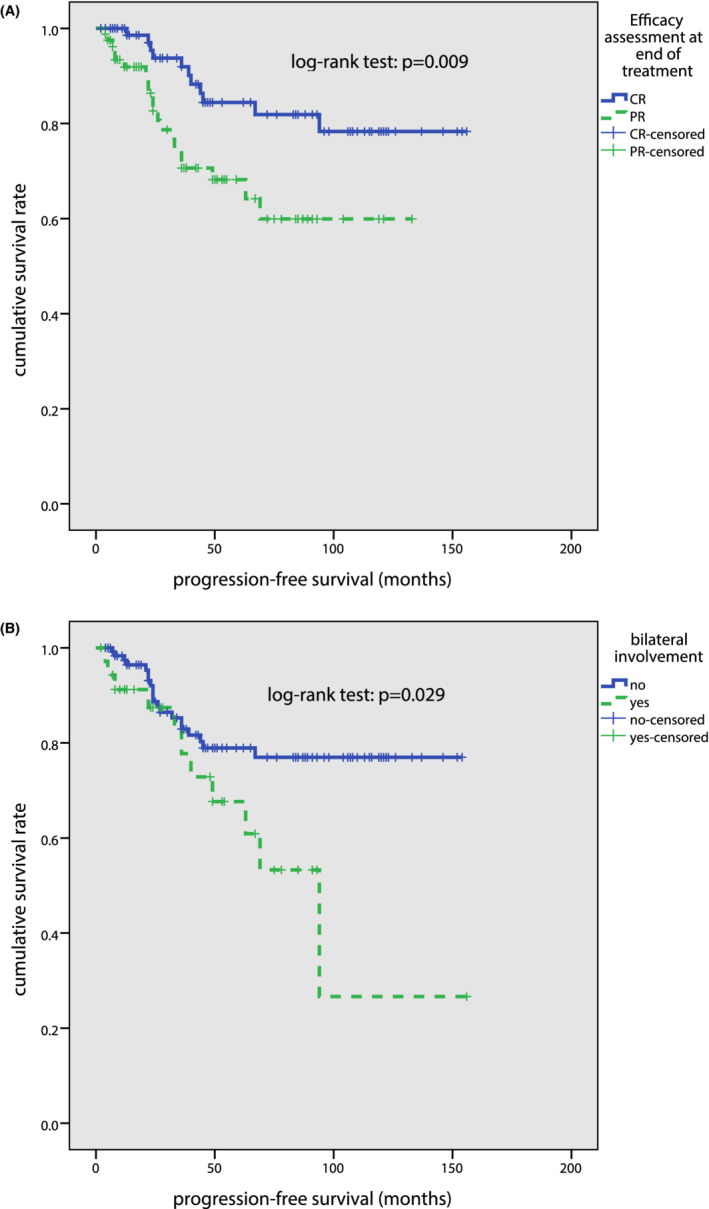
Univariate survival analysis for patients with primary OAML. (A) Progression‐free survival (PFS) curves among patients with CR or PR at the end of treatment. (B) Progression‐free survival (PFS) curves among patients with unilateral involvement or bilateral involvement.

Further analysis of the correlation between clinical features and prognosis revealed that unilateral/bilateral involvement was associated with PFS. Patients with CR at the end of treatment had significantly better PFS than those with PR (*p* = 0.009; Figure 4A). Patients with bilateral involvement (*p* = 0.029; Figure 4B) at diagnosis was associated with significantly inferior PFS. Moreover, age, IPI score, or TNM staging were not associated with PFS. None of these clinical factors were found to be associated with OS.

### 3.4 Side effects of further treatment

Of the 30 patients that received radiotherapy, alone or in combination with chemotherapy, 15 suffered from conjunctivitis and eight had localized skin edema. All these symptoms disappeared within six months after radiotherapy. Three patients who received a dose of more than 3600 cGy radiotherapy developed cataract, while none of them received cataract surgery during follow‐up. Five patients were diagnosed with xerophthalmia within six months after radiotherapy. Of the 31 patients that received chemotherapy, alone or in combination with radiotherapy, 35.5% of patients had grade I–II gastrointestinal toxicity and 3.2% had grade II hepatotoxicity. Concerning hematologic side effects, 22.6% had grade I–II neutropenia, 12.9% had grade I‐II thrombocytopenia, 6.5% had grade I–II anemia, and 3.2% had grade III neutropenia. None of the patients in the whole cohort died of treatment‐related toxicities.

## 4 DISCUSSION

Using retrospective data of 166 primary OAML patients in a single institution in Beijing, China, we found that bilateral involvement was associated with reduced 5 and 10‐year PFS. We also found that, among patients with residual lesions after surgical treatment, further consolidation treatment with radiotherapy, chemotherapy, or combined radio‐ and chemotherapy was associated with improved survival, compared with patients in the watchful waiting group. Finally, we showed that, although rare, patients with primary OAML might experience late disease progression or relapse more than five years after treatment.

OAML is a subtype of malignancy of the ocular adnexal, accounting for 55% of all ocular tumors.^1^ MALT is the most common pathological subtype of ocular adnexal lymphomas, accounting for 38–98% of ocular adnexal lymphoma.^12^, ^13^ An increasing incidence of OAML has been reported recently, with higher incidence reported in Asian countries than Western countries.^14^ Zhang et al explored the relationship between *Chlamydia psittaci* and OAML in northern China, and none of the patients had *Chlamydia psittaci* infection.^15^ Thus, we did not routinely examine the possible pathogens in our cohort. Men have a higher incidence of OAML than women, especially in Asian populations, and the median age of diagnosis ranges from early 40 to 70 s.^2^, ^3^, ^16^ In the present study, 58.4% of the patients were male and the median age of diagnosis was 57 years old. One previous study demonstrated that patients with OAML did not show B symptom and the proportion of patients with increased level of LDH was less than 10%.^17^ In agreement with this, we found one patient with B symptom and three patients with elevated levels of LDH in the present study. Our results further demonstrated the in general indolent disease course of MALT. Patients with primary OAML usually have unilateral lesion, and orbit is known as the most common site of involvement, followed by conjunctiva (35–40%), lacrimal gland (10–15%), and eyelid (<10%).^1^, ^2^, ^3^ In the present study, we found 78.3% patients with unilateral involvement, 28.3% patients with orbital involvement, and 37.4% patients with involvement of multiple sites.

There is currently no consensus about optimal treatment strategies for OAML.^18^, ^19^, ^20^ The purpose of surgical excision is, on the one hand, to make clear diagnosis through collecting pathological samples from surgery and, on the other hand, to treat the disease through removing lesions. Patients with residual lesions after surgery, that is, no complete resection of neoplasm, could choose watchful waiting or further treatment, according to involvement of lesions as well as the general health status and willingness of the patient. The response rate of initial treatment was estimated as 95%–100%, regardless of the type of treatment.^2^, ^3^, ^21^ There is however still a high risk of local and distant relapse, with a relapse rate ranging from 14.4% to 48%.^19^, ^22^, ^23^, ^24^, ^25^ Sueng et al. studied in 198 patients with OAML that received radiotherapy with a dose of 30 (20–45) Gy and found a response rate of 96.15% and a relapse rate of 14.14%.^20^ Sung Yong et al. studied in 84 patients with bilateral involvement that received bilateral radiotherapy with a dose of 27 (20–40) Gy and found a complete remission rate of 80.9%, a partial remission rate of 16.7%, a 10‐year PFS rate of 79.8%, a 10‐year OS rate of 91.1%, and 11 patients underwent a cataract surgery during follow‐up.^26^ A study in South Korea used R‐CHOP/R‐CVP as the first‐line treatment of localized OAML and found an overall response rate of 100%.^17^ Among the enrolled 33 patients, they found three cases with relapse and a 4‐year PFS rate of 90.3%. In our study, 53 patients received further treatment with a complete remission rate of 64.2%, a partial remission rate of 35.8%, an overall response rate of 100%, and a disease progression or relapse rate of 11.3% during a median follow‐up of five years. When focusing on patients with residual lesions after surgery, patients receiving further consolidation treatment had longer PFS compared with patients with watchful waiting, suggesting that additional non‐surgical intervention could further reduce the risk of disease progression and prolong PFS. For patients with complete tumor resection, it is still uncertain whether consolidation with rituximab alone or rituximab‐based immunochemotherapy would improve PFS or not. For patients of follicular lymphoma who have high baseline tumor burden, rituximab consolidation for 2 years has been confirmed to provide PFS benefits.^27^ However, considering the relatively more indolent behavior and low tumor burden of OAML, it is difficult to benefit from rituximab consolidation. Whether rituximab consolidation would reduce relapse rate of patients with bilateral eye involvement or not needs to be explored prospectively.

Previous studies demonstrated that patients with localized lesions could achieve favorable prognosis through localized radiotherapy, whereas systemic treatment, such as chemotherapy, immunotherapy, and combined radiotherapy with chemotherapy, is more likely to be used for patients with distant metastasis. In recent years, however, systemic treatment, especially immunochemotherapy, is increasingly commonly used for patients with localized lesions. In our study, no statistical difference in PFS was found among patients with different consolidation strategies. However, the treatment toxicity profiles were different among those patients. Side effects caused by radiotherapy, including cataract and xerophthalmia, are being increasingly recognized as significantly reducing quality of life, especially for young patients. A study of 79 patients with conjunctival OAML in South Korea found that the incidence of cataract, xerophthalmia, and ophthalmalgia was 6.3%, 26.6%, and 5.1%, respectively, after radiotherapy.^28^A Japanese study summarized data from 46 patients of ocular adnexal lymphomas and found that up to 30% of these patients underwent cataract surgery after radiotherapy.^20^, ^29^Sung Yong et al. conducted a study of R‐CVP regimen for the treatment of patients with limited‐stage OAML with bilateral or beyond‐conjunctival involvement and reported a 4‐year PFS rate of 90.3% and a 4‐year OS rate of 100%.^17^ All patients completed six cycles of R‐CVP, followed by two cycles of rituximab therapy, without dose adjustment, and only a third of patients experienced neutropenia and peripheral neuropathy. In the present study, we did not observe serious side effects among patients with systemic treatment, apart from bone marrow suppression, gastrointestinal reaction, etc., all of which resolved at the end of treatment. If verified in future studies, systemic treatment might be a safe and effective treatment for OAML with limited lesions. However, due to the retrospective nature of our study, several limitations exist. Firstly, all patients visited our Eye Center initially, and some of them lost follow‐up after complete surgical resection; secondly, none patients in our cohort received rituximab alone; thirdly, various numbers of chemotherapy regimens were used and it is difficult to draw a robust recommendation on the optimal treatments. Thus, it is of urgent need to conduct prospective randomized clinical trials to define the role of surgery, radiation, and immunochemotherapy.

In conclusion, we found that bilateral involvement was associated with a poor prognosis of OAML and call for extended surveillance of disease progression for patients with bilateral involvement. Among patients with residual lesions after surgical treatment, further consolidation treatment could lead to improved survival, compared with watchful waiting. Systemic treatment might be considered as a treatment candidate, given the noted rare adverse event.

## AUTHOR CONTRIBUTION

LW and JMM conceived the study and treated all patients. YZL, RYF, XLL, and XDL collected and analyzed the data. YSP confirmed the pathologic diagnosis. YZL and LW wrote the paper. All authors revised the paper and approved the final submission of the paper.

## FUNDING INFORMATION

This work was supported by grants from National Natural Science Foundation of China (grant No. 81873450, 82170181), Beijing Municipal Natural Science Foundation (grant No. 7222027), and Beijing Municipal Administration of Hospitals' Youth Programme (code: QMS20200201) to Liang Wang.

## CONFLICT OF INTEREST

No conflicting relationship exists for any author.

## Data Availability

The data that support the findings of this study are available from the corresponding author upon reasonable request.
